# Association between sedentary behaviour and risk of dementia: an evidence gap

**DOI:** 10.1038/s41398-021-01316-8

**Published:** 2021-03-30

**Authors:** Kirsten Dillon, Paul A. Gardiner

**Affiliations:** 1 The University of Western Ontario, London, Canada; 2 The University of Queensland, Brisbane, Australia

**Keywords:** Diseases, Scientific community

Dear Editor,

Given the lack of a cure, it is important to investigate risk factors for dementia to target prevention initiatives. A study recently published in Translational Psychiatry by Yan et al.^1^ examines the association between sedentary behaviour [sic] and the risk of dementia. However, the authors have misused the term sedentary behaviour, which misrepresents their findings and leads to a false conclusion. Historically, sedentary behaviour was used to denote lack of physical activity. Now ‘sedentary behaviour’ is defined as “any waking behaviour characterized by energy expenditure ≤1.5 metabolic equivalents while in a sitting, reclining or lying posture”^2^. In contrast, the term ‘physical inactivity’ is defined as “an insufficient physical activity level to meet present physical activity recommendations”^2^. For example, the World Health Organization (WHO) states the global physical activity guidelines as 150 min of moderate-intensity, or 75 min of vigorous-intensity aerobic physical activity a week (WHO, 2020^3^). As many people in today’s society are both physically inactive and sedentary^4^, it is imperative that we differentiate between these two terms.

Ten years ago, Owen and colleagues clearly defined and differentiated the various health consequences of too much sitting (sedentary behaviour) versus physical inactivity^5^. In 2012, the Sedentary Behaviour Research Network (SBRN) was aware of the inconsistencies in the terminology around sedentary behaviour and proposed a formal definition of sedentary behaviour, which was updated in 2017 (see above). Given the increasing evidence on the deleterious associations of sedentary behaviour with health outcomes^6^, the Sedentary Behaviour Council of the International Society of Physical Activity and Health successfully advocated for the (United States) National Library of Medicine to create a sedentary behaviour Medical Subject Heading (MESH) term^7^. Without the proper definition and use of these terms going forward, future research will be confused by their inappropriate use.

Sedentary behaviour research is well established with evidence accumulating for over two decades on deleterious associations with health outcomes such as a higher risk of type 2 diabetes, cardiovascular disease (fatal and non-fatal), and all-cause mortality^6^. More nascent evidence is emerging on the relationship of sedentary behaviour with cognitive function. Since 2016, there have been four systematic reviews published indicating mixed associations between various sedentary behaviours and cognitive function^8–11^. The first review by Falck et al.^9^ indicated that higher levels of sedentary behaviour are associated with lower cognitive function. Copeland et al.^8^ then indicated that the association may vary depending on the domain of sedentary behaviour being assessed. Finally, Loprinzi^10^ and Olanrewaju et al.^11^ stated an overall lack of clarity in the association of sedentary behaviour with cognitive function. Overall, although there was a slight trend towards more sedentary behaviour being associated with worse cognitive function, none of these reviews were able to distinguish a clear association of sedentary behaviour with the risk of dementia. Therefore, a gap remains, which was why we were encouraged to read this article by Yan et al.^1^. However, upon further investigation of the article, it became clear that it does not address this association, and thus, the gap still remains. More specifically, the article fails to properly define ‘sedentary behaviour’ as the exposure variable. As a result of this, none of the studies included within the systematic review and meta-analysis by Yan et al.^1^ were in fact reporting ‘sedentary behaviour’ as the exposure variable but rather physical inactivity (see Table 1). Hence, it is recommended that the authors revise their manuscript to remove any mentions of sedentary behaviour and replace them with ‘physical inactivity’. This would result in the conclusion that ‘physical inactivity’ is significantly associated with an increased risk of dementia, while the association of ‘sedentary behaviour’ with dementia still warrants further investigation. To address this gap, future studies should use the definition of sedentary behaviour supported by SBRN and use devices, e.g., activPAL inclinometer (http://www.palt.com) in conjunction with a subjective questionnaire to capture the context and specific types of sedentary behaviours. This will allow us to quantify the risk of sedentary behaviour on development of dementia in meta-analysis and also identify any differences in sedentary behaviour in people with and without dementia. Given the often-long time frame to develop dementia, interventions should consider using proxy measures such as cognitive function to assess their impact on health outcomes.

**Table 1:** Description of exposure variables used within each study of Yan et al.’s^1^ meta-analysis.

Study	Exposure variable
Deckers et al. (2018)	Physical inactivity: No exercise or physical activity in the last week.
Kishimoto et al. (2016)	Physical inactivity: No reported leisure time exercise in the last week.
Luck & Riedel-Heller (2016)	Physical inactivity: Proportion of the adult population that self-report not participating in sports.
MacDonald et al. (2015)^a^	Physical inactivity: No definition.
Luck et al. (2014)	Physical inactivity: Participating in an activity less than or equal to once a week, or never.
Mehlig et al. (2014)	Physical inactivity: Less than 4 h of leisure time physical activity.
Norton et al. (2014)	Physical inactivity: Do not do either 20 min of vigorous activity on 3 or more days or 30 min of moderate activity on 5 or more days per week.
de Bruijn et al. (2013)	Physical activity: Hours spent on activities such as walking, cycling, gardening, diverse sports, hobbies and housekeeping activities.
Verdelho et al. (2012)	Physical inactivity: Not participating in at least 30 min of activity on at least 3 days per week.
Gelber et al. (2012)	High physical activity: Defined as the highest quartile of daily hours spent in slight or moderate activity (e.g., walking, gardening, carpentry). Low physical activity: Sleeping, lying, sitting or standing.
Keller et al. (2010)^a^	Physical inactivity: No activity with a physical component reported.
Scarmeas et al. (2009)	Physical activity: Time spent on a series of vigorous, moderate and light activities summed into an overall physical activity score, which was then categorized into tertiles: no physical activity (used as reference), some physical activity and much physical activity.
Kivipelto et al. (2008)^a^	Physical inactivity: Not participating in leisure time physical activity less than two times a week.
Rovio et al. (2005)	Physical inactivity: Participating in leisure-time physical activity less than twice a week.
Rovio et al. (2007)^a^	Physical inactivity: Answering “no” to how many minutes do you walk, bicycle or have some other physical activity when you are going to and from work?
Anttila et al. (2002)	Physical inactivity: Not holding one of the following ‘sedentary’ occupations: office work, intellectual work and service branch work.
Laurin et al. (2001)	Physical activity: Engaging in exercise three or more times per week in an intensity greater than or equal to walking. Physical inactivity: All other combinations of frequency and intensity.
Yoshitake et al. (1995)	Physical inactivity: No daily exercise during the leisure period or moderate to severe physical activity at work.

^a^Studies not included in the reference list of the paper, but found by manual searching using author, year and dementia as a search term.
